# *Hypericum japonicum* extract inhibited porcine epidemic diarrhea virus *in vitro* and *in vivo*

**DOI:** 10.3389/fphar.2023.1112610

**Published:** 2023-04-17

**Authors:** Hongyu Rao, Weiwei Su, Xiaoli Zhang, Yonggang Wang, Tao Li, Jinliang Li, Xuan Zeng, Peibo Li

**Affiliations:** Guangdong Engineering and Technology Research Center for Quality and Efficacy Reevaluation of Post-Market Traditional Chinese Medicine, Guangdong Key Laboratory of Plant Resources, State Key Laboratory of Biocontrol, School of Life Sciences, Sun Yat-sen University, Guangzhou, China

**Keywords:** *Hypericum japonicum*, porcine epidemic diarrhea virus, antiviral effect, watery diarrhea, intestinal microbiota, piglets

## Abstract

Porcine epidemic diarrhea virus (PEDV) infection causes lethal watery diarrhea and high mortality in neonatal piglets, leading to huge economic losses in the global swine industry. Currently, the existing commercial vaccines cannot fully control PEDV, so it is urgent to develop effective antiviral agents to complement vaccine therapy. In the present study, we investigated the antiviral effect of *Hypericum japonicum* extract (HJ) against PEDV *in vivo* and *in vitro*. In *in vitro* assays, HJ could directly inactivate PEDV strains; moreover, it inhibited the proliferation of PEDV strains in Vero or IPI-FX cells at its non-cytotoxic concentrations. Time of addition assays revealed that HJ mainly inhibited PEDV at the later stages of the viral life cycle. In *in vivo*, compared with the model group, HJ could reduce the viral titers in the intestines of infected piglets, and improve their intestinal pathological, indicating that HJ could protect the newborn piglets from highly pathogenic PEDV variant infection. Furthermore, this effect may be related to the fact that HJ can not only directly inhibit viruses, but also regulate the structure of intestinal microbiota. In conclusion, our results indicate that *Hypericum japonicum* could inhibit PEDV replication *in vitro* and *in vivo* and might possess the potential to develop as the anti-PEDV drug.

## 1 Introduction

Porcine epidemic diarrhea (PED) is a highly contagious enteric disease in piglets caused by porcine epidemic diarrhea virus (PEDV), which are associated with symptoms, including vomiting, acute diarrhea, and dehydration (Cai et al., 2019; Jung et al., 2020). PEDV, which belongs to the genus *Alphacoronavirus* of the coronavirus family, can infect pigs of all ages and has a high mortality rate especially in suckling piglets less than 10 days of age, making it one of the most serious diseases in the swine industry (Stadler et al., 2018; Wang Q. et al., 2019). PEDV was first reported in Europe in the 1970s, and then quickly spread to Asia and other regions (Wood, 1977; Sun et al., 2012). At first, the incidence and mortality of PED were not high. However, the new PEDV variants have emerged in China since late 2010, and frequent outbreaks have occurred in Europe, Asia, and America, resulting in huge economic losses (Li et al., 2012; Wang et al., 2013). Despite the widespread availability of PEDV vaccines, recent studies indicate that ongoing variation of the S1 gene is occurring in the PEDV strains, making that PED recurrence has become more common in pigs immunized with commercial vaccines (Su et al., 2020). Therefore, it is urgent to develop anti-PEDV therapeutic drugs. Traditional Chinese medicine (TCM) can exhibit anti-PED effects from multiple targets and perspectives, such as virus inhibition, anti-inflammation, and immune regulation, which is one of the best ways to develop new drugs against PED (Chen et al., 2020; Ding et al., 2020; Xu et al., 2020; Liu et al., 2021). Therefore, this study focuses on the potential application of TCM in PED treatment.

*Hypericum japonicum* Thunb. ex Murray is a species of herbaceous plant of *Hypericum Linn* in Gambogic, which is widely distributed in Asia. In traditional Chinese medicine theory, *Hypericum japonicum* (HJ) has been used for jaundice, swelling, and alleviating internal heat for a long time. While modern pharmacological experiments have proved that *Hypericum japonicum* possessed multiple biological activities, such as anti-inflammatory, antioxidant, antiviral, and healing of liver injury (Huang M. et al., 2018). One research study found that *Hypericum japonicum* exhibited its anti-influenza virus effects by significantly extending the survival time of influenza A (H3N2)-infected mice and inhibiting the lung consolidation of mice infected with pneumonia (Liu et al., 2008). Another study suggests that xanthones and flavonoids derived from *Hypericum japonicum* show potential beneficial effects in the treatment of coronavirus disease 2019 due to binding multiple receptors, such as converting enzyme (ACE2) and 3CL protease (Mpro) (Su et al., 2021). In addition, it has been reported that *Hypericum japonicum* inhibited the necrosis and degeneration of liver cells and inflammatory infiltration induced by duck hepatitis B virus (Li et al., 2011). These research studies suggest that *Hypericum japonicum* may have a broad-spectrum antiviral effect and possess an excellent prospect to develope as a new drug in the treatment of viral infectious diseases caused by viruses. However, the detailed information about the antiviral capacity of *Hypericum japonicum* against PEDV remains unclear. Therefore, in this study, we investigated the antiviral activity of the *Hypericum japonicum* extract *in vitro* using Vero or IPI-FX cells infected with different PEDV strains. In addition, the *in vivo* antiviral effect of the HJ was studied in the newborn piglets infected with PEDV-G2 strain as well. Our result suggested that HJ could inhibit PEDV infection *in vitro* and *in vivo*, indicating that *Hypericum japonicum* might be used to develop as anti-PEDV therapeutics.

## 2 Materials and methods

### 2.1 Cells and virus

The African green monkey kidney (Vero) cell line and the immortal pig intestinal (IPI-FX) cells were kindly provided by Dr. Cao Yongchang (Sun Yat-sen University, China). All cells were grown in Dulbecco’s modified Eagle’s medium (DMEM supplemented with 10% inactivated fetal bovine serum (FBS) (Gibco, United States). The PEDV-CV777 stain was obtained from Cao’s lab, while the PEDV-G2 strain was isolated and reported by Dr. Gong Lang (South China Agricultural University, China). The maintenance medium for PEDV propagation was DMEM supplemented with 7.5 μg/mL trypsin (Gibco, United States). Working stocks of all the PEDV stains were prepared by Vero cells. Briefly, Vero cells were infected with the PEDV and incubated for 1–2 days, monitoring the development of cytopathic effect (CPE) daily until CPE ≥80% of cell monolayer. Then, the supernatant was transferred at −80°C. After three cycles of freezing and thawing, lysates were transferred to sterile 50-mL conical tubes and centrifuged at 12,000 rpm. Then, the supernatants were aliquoted and stored at −80°C until use. Virus titers were detected by using the Reed–Muench method and expressed as tissue culture infective dose 50% (TCID_50_). The plaque-forming unit (PFU) was determined according to “PFU = 0.7 × TCID_50_,” and the multiplicity of infection (MOI) was calculated based on the PFU.

### 2.2 Drugs and reagents

*Hypericum japonicum* used in the experiments were purchased from Wuzhou, Guangxi Province, China. In addition, the identification of plants was performed by Dr. Liao Wenbo (Sun Yat-sen University, China). The dry whole plant of *Hypericum japonicum* was taken, washed to remove the weeds and soil of the grass, and sliced into pieces of about 1–2 cm. About 500 g of *Hypericum japonicum* were extracted with 6 L of deionized water for 0.5 h each time to extract three times. The extracts were collected separately and then concentrated by rotary vaporization at 60°C under reduced pressure. The mixture filtrate was collected and stored at 4°C. All extracts were diluted with DMEM (Gibco, United States).

Ribavirin was purchased from Shanghai Yuanye Bio-Technology Co., Ltd., China, and dissolved in DMSO to make a solution of 100 mg/mL and diluted 1,000 times with DMEM.

### 2.3 Cytotoxicity assay

Cell viability of Vero and IPI-FX cells was determined using commercial cell counting kit-8 (CCK-8) (Dojindo, Shanghai) in accordance with the manufacturer’s instructions. Briefly, cells were spread on 96-well plate and cultured for 80% confluent, and then incubated with or without HJ for 24 h and 48 h, respectively. After the cells were washed twice with sterile pH 7.4 phosphate buffered saline (PBS, 1×) (Gibco, United States), 10 μL of CCK-8 reagent and 90 μL of DMEM were added to each well and incubated at 37°C for 1 h. The CCK-8 signal was measured at an absorbance of OD_450nm_. The relative viability of the cells was calculated as the percentage of the optical density relative to that of the control sample.

### 2.4 Inhibition of virus infection

Vero cells were incubated with DMEM or various concentrations of drugs for 1 h in 12-well plate or 24-well plate, following by infection with PEDV (MOI = 0.1) for 2 h, and then the virus inoculum was removed and fresh DMEM containing different concentrations of HJ was added. Cells were collected at 24 h to determine whether any change had occurred in the PEDV nucleocapsid (N) protein by western blot assay, and whether any change had occurred in the PEDV N gene by quantitative reverse–transcriptase PCR (qRT-PCR) assay, respectively. In addition, the inhibitory effect of drugs on viral proliferation was determined by using TCID_50_ analysis.

### 2.5 TCID_50_ analysis

The cells were seeded into a 96-well plate for 24 h. The solution was removed, and monolayers of cells were washed twice with PBS. Then, 10-fold series dilution of viral samples was added to the 96-well plate. Each group was diluted in 8 duplicate wells and cultured at 37°C for 3–5 days. Immunofluorescence assay was conducted to observe and record PEDV-infected cells. Briefly, cells were fixed with 4% paraformaldehyde (Dalian Meilun Biotech Co., Ltd., Dalian) for 15 min and then permeabilized with 0.2% Triton X-100 (Dalian Meilun Biotech Co., Ltd., Dalian) for 10 min at room temperature. After the permeabilization treatment, cells were blocked with 1% bovine serum albumin (BSA) (Beijing Solarbio Science and Technology Co., Ltd., Beijing) and then stained overnight at 4°C with anti-PEDV N polyclonal antibody diluted with 1:10000 (Xu, et al., 2020). After being washed with PBST three times, cells were incubated with goat anti-mouse secondary antibody (Abcam, Shanghai) diluted with 1:10000 labeled with fluorescein isothiocyanate (FITC) at 37°C for 1 h. The fluorescent particles were observed and counted using a fluorescence microscope (Leica DMi8, Germany).

### 2.6 qRT-PCR analysis

Total RNA from Vero and IPI-FX cells was extracted using an RNA easy kit. (EZBioscience, China) according to the manufacturer’s instruction. RNA concentration was determined using NanoPhotometer-N60, while about 200 ng of total RNA was used for cDNA by reverse transcription using an RT-PCR kit (Nanjing Vazyme Biotech Co., Ltd., China). The real-time qPCR was carried out with GoTaq^®^ qPCR Master Mix (Promega, United States) on a LightCycler 480 (Roche, Mannheim, Germany), as recommended by the manufacturer. The cycling parameters were as follows: 95°C for 10 min; 45 cycles of 95°C for 10 s, 60°C for 20 s, and 72°C for 20 s; and 1 cycle of 95°C for 5 s and 65°C for 1 min. The specific primers purchased from Sangon Biotech Co., Ltd. (Shanghai, China) were as follows:

PEDV N gene forward (5′-GAA​AAT​CCT​GAC​AGG​CAT​AAG​CA-3′); PEDV N gene reverse (5′-TTG​CCG​CTG​TTG​TCA​GAC​TT-3′);
glyceraldehyde-3-phosphate dehydrogenase (GAPDH) forward (5′-CCT​TCC​GTG​TCC​CTA​CTG​CCA​AC-3′);
GAPDH reverse (5′-GAC​GCC​TGC​TTC​ACC​ACC​TTC​T-3′).
Fold change in gene expression was calculated using the 2^−ΔΔct^ method, and all PCR reactions were performed in triplicate.

### 2.7 Western blot analysis

Vero cells or IPI-FX cells were washed with 1 mL of ice-cold PBS three times. The cells were lysed in radio immunoprecipitation assay (RIPA) (Shanghai Beyotime Biotechnology Co., Ltd., Shanghai) lysis buffer supplemented with 1% protease inhibitors (Yataihengxing, China). After centrifugation, the supernatant was boiled with 5× SDS (Shanghai Beyotime Biotechnology Co., Ltd., Shanghai) loading buffer for 7 min. Equivalent volume of protein samples was fractionated by 10% sodium dodecyl sulfate-polyacrylamide gels (SDS-PAGE) electrophoresis (Bio-Rad, United States) and then transferred onto polyvinylidene fluoride (PVDF) (Bio-Rad, United States) membranes. After being washed in the Tris-buffered saline with Tween (TBST) (GenStar, Canada), membranes were blocked with 5% skim milk (Becton, Dickinson and Company, United States) and then incubated for overnight at 4°C with mouse monoclonal antibody against PEDV N protein (Beijing Biolead Biology Sci. & Tech. Co., Ltd., Beijing, China) (1:500) or mouse monoclonal antibody against GAPDH (Abcam, United States) (1:10,000). The blots were subsequently incubated with HRP-conjugated goat anti-mouse IgG (1:10,000) for 1 h at room temperature. The blots were visualized using enhanced chemiluminescence (ECL) (Bio-Rad, United States), and Tanon5200. The densitometric analysis was quantified using ImageJ, and all determinations were performed in triplicate.

### 2.8 Time of addition assay

IPI-FX cells were seeded on a 12-well plate and cultured overnight before infection with PEDV. In short, IPI-FX were inoculated with PEDV G2 (MOI = 0.4) at 4°C for 1 h. After inoculation, cells were washed with cold PBS three times, then 1.5 mL of maintenance medium was added to each well, and all samples were placed in a humidified incubator at 37°C. Meanwhile, the processing methods of PEDV incubated with HJ from 1 to 12 hours post infection (hpi) were classified into M0–M7. Briefly, IPI-FX cells only incubated with HJ was set as mock (M0), while IPI-FX cells only infected with PEDV were used as model M1. M2 was set as positive control by 1–12 hpi. M3 was set as pretreatment by 1–0 hpi. M4 was set as adsorption by 0–1 hpi. M5 was set as invasion by 1–2 hpi. M6 was set as both adsorption and invasion by 0–2 hpi. M7 was set as replication by 2–12 hpi. After 12 h, the protein levels of N in cells were measured by western blot assay.

### 2.9 Animal experiments

All the procedures used in this experiment have been approved by the Animal Experimental Committee of South China Agricultural University (Guangdong, China) (certification no. SCAU-2021C083). Fourteen crossbred (Duroc × Landrace × Big White) conventional newborn piglets were procured from Xinyi Xianshun Agricultural Development Co., Ltd. (Guangdong, China). After confirming that all piglets were not infected with PEDV by the Animal Disease Detection and Diagnosis Center of South China Agricultural University, the piglets were randomly divided into two groups (seven piglets per group). Group 1 represented the model group, and group 2 represented the treated group. All piglets were housed in one room and fed with a mixture of skim milk powder (Beijing precision animal nutrition research center, China) with warm water no less than five times a day before the experiment. At 3 days of age, the piglets in group 2 were orally given HJ at 1.28 g/kg body weight, twice a day, while the group 1 received water at the same time. After 6 days of administration, all piglets were orally challenged with 5 mL DMEM containing a total of l0^5^ PFU of PEDV-G2 solution. Dead piglets were recorded and immediately necropsied and sampled. All piglets were euthanized 48 h after infection, and their ileum tissues were collected for the detection of virus content and histopathology staining (three piglets were randomly selected for histopathology staining from each group). Meanwhile, the ileum contents were collected and then stored at −80°C until DNA extraction.

### 2.10 Intestinal microbiota analysis

Genomic DNA was extracted from ileum contents using the TGuide S96 Magnetic Soil/Stool DNA Kit [Tiangen Biotech (Beijing) Co., Ltd.] according to the manufacturer’s instructions. Furthermore, the Qubit dsDNA HS assay kit and the Qubit 4.0 fluorometer (Invitrogen, Thermo Fisher Scientific, Oregon, United States) were used to determine the quality and concentration of DNA. Next-generation sequencing library preparations were conducted at Biomarker Technologies Co., Ltd. (Beijing, China). Briefly, a panel of proprietary primers was designed to amplify the relatively conserved regions bordering the V3 and V4 hypervariable regions using forward primers containing the sequence “AGRGTTTGATYNTGGCTCAG” and reverse primers containing the sequence “TASGGHTACCTTGTTASGACTT.” Then, the total PCR amplicons were purified with Agencourt AMPure XP Beads (Beckman Coulter, Indianapolis, IN) and quantified using the Qubit dsDNA HS assay kit and the Qubit 4.0 fluorometer (Invitrogen, Thermo Fisher Scientific, Oregon, United States).

Amplicons were pooled in equal quantities after the individual quantification step. Afterward, the SMRTbell libraries were prepared from the amplified DNA using SMRTbell Express Template Prep Kit 2.0 according to the manufacturer’s instructions (Pacific Biosciences). The purified SMRTbell library combined with primer and polymerase was placed on the Sequel II Sequencing kit 2.0.

Sequence data were analyzed on BMK Cloud (Biomarker Technologies Co., Ltd., Beijing, China). Briefly, SMRT Link software (version 8.0) was used to screen the original reads generated from sequencing in order to obtain the circular consensus sequencing (CCS) reads. Subsequently, the CCS containing no primers and those reads beyond the length range (1,200–1,650 bp) were discarded through the quality filtering using the Cutadapt quality control process (version 2.7). The clean reads obtained from the UCHIME algorithm (v8.1) were conducted on feature classification to output amplicon sequence variants (ASVs) using the divisive amplicon denoising algorithm (data 2), and the ASVs with reabundance <0.005% were filtered. The Ace, Chao1, Simpson, and Shannon index estimates of alpha diversity were calculated and shown using the QIIME2 and R software, respectively. Principal coordinate analysis (PCoA), heat map, UPGMA, and non-metric multidimensional scaling (NMDS) were used to analyze the beta diversity and then to evaluate the degree of similarity of microbial communities from different samples using QIIME.

### 2.11 Statistical analysis

Statistical comparisons were performed using GraphPad Prism software. Accordingly, the significance of the differences between the treatment group and the control (cells viability, PFU, and N mRNA) was determined by the ANOVA and student's t test, and the survival curve was analyzed using the logrank test.

## 3 Results

### 3.1 Evaluation of the cytotoxicity of HJ

**FIGURE 1:**
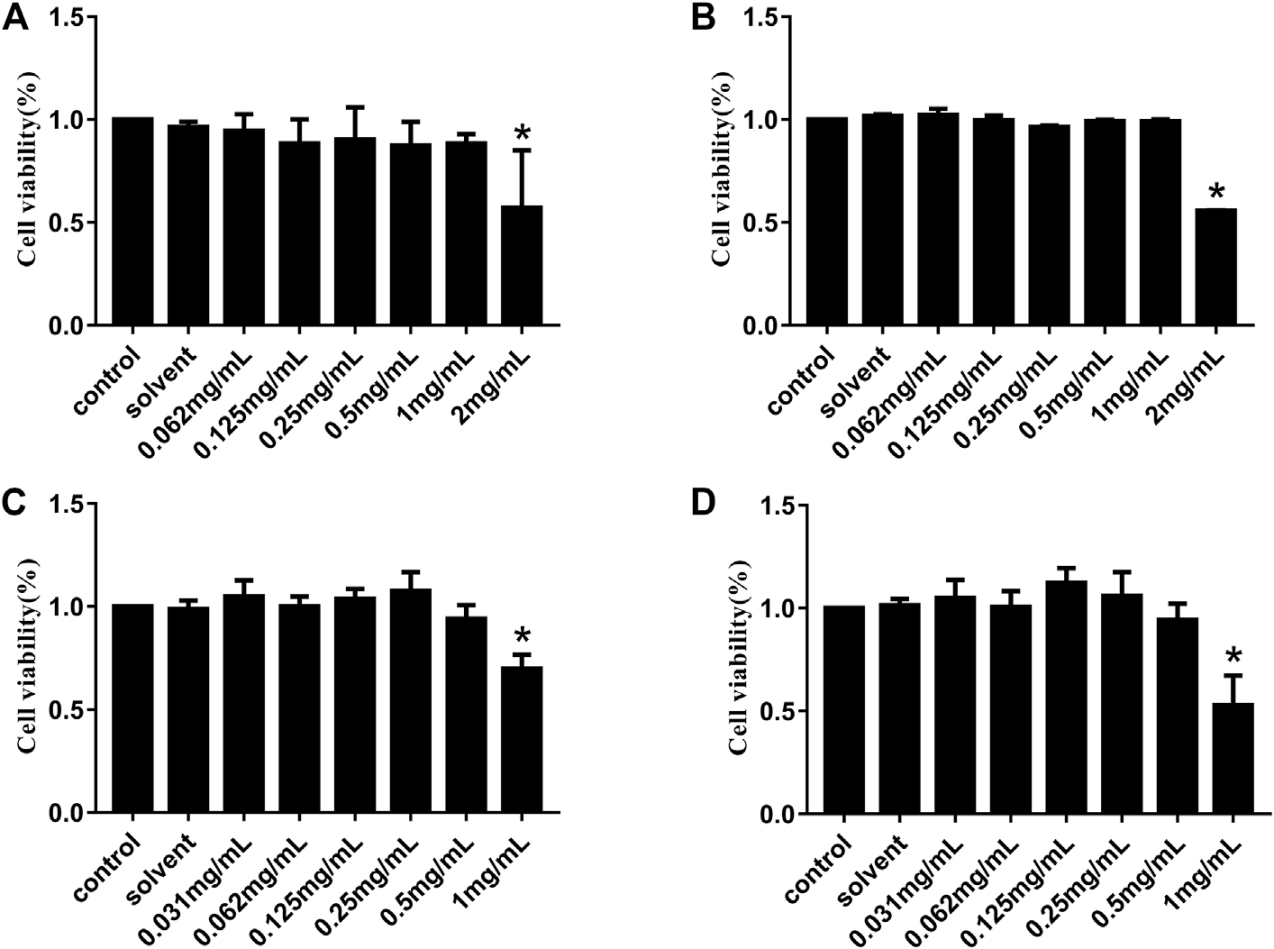
Cytotoxicity of HJ to Vero and IPI-FX cells *in vitro*. Viability of Vero cells treated with different concentrations of HJ (0.062–2 mg/mL) for 24 h **(A)** and 48 h **(B)**, and the viability of IPI-FX cells treated with different concentrations of HJ (0.031–1 mg/mL) for 24 h **(C)** and 48 h **(D)** were measured by CCK-8 method. Meanwhile, the control normal DMEM or solvent DMEM with sterile water for 24 h and 48 h were detected. Results are representative of three independent experiments. Data are represented as mean ± SD, *n* = 3. *, and *p* < 0.05 was considered statistically significant compared with the control group.

The CCK-8 assay was used to detect the relative viability of Vero and IPI-FX cells treated with HJ so as to explore the potential cytotoxicity of HJ. Vero or IPI-FX cells were cultured with different concentrations of HJ for 24 h and 48 h, respectively. The results in Figure 1 showed that compared with the control, the relative survival rates of the Vero cells at 24 h and 48 h were decreased at 2 mg/mL HJ, while there was no significant difference in the relative survival rate of 1 mg/mL, suggesting that HJ at 1 mg/mL had good biocompatibility with the Vero cells. The results of IPI-FX cells treatment with HJ showed that 0.5 mg/mL of HJ had good biocompatibility for IPI-FX cells. Therefore, the dosing of HJ was 1 mg/mL on Vero cells and 0.5 mg/mL on IPI-FX cells.

### 3.2 HJ inhibited PEDV infection *in vitro*

**FIGURE 2:**
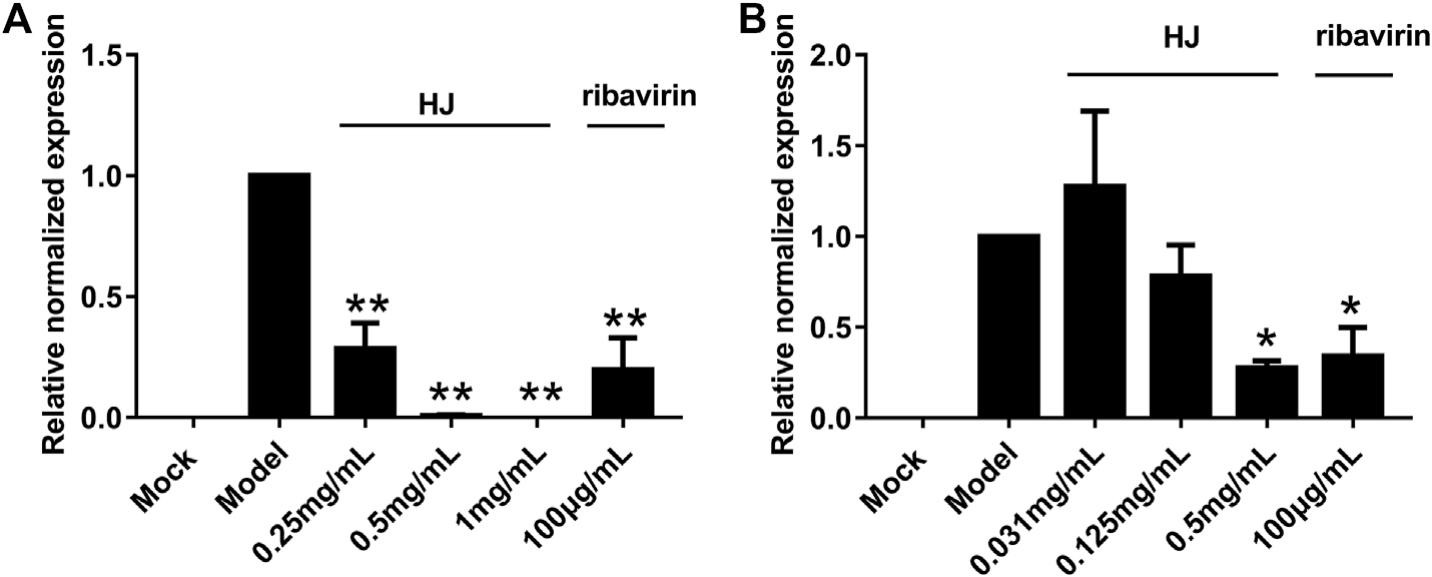
The PEDV N gene in infected Vero **(A)** or IPI-FX cells **(B)** was examined with real-time PCR using specific primers. Cells were treated with HJ throughout the experiment. The expression level of N mRNA was calculated in relation to the expression level of GAPDH at 24 hpi. Results are representative of three independent experiments. Data are represented as mean ± SD, *n* = 3. *, and *p* < 0.05; **, and *p* < 0.01 were considered statistically significant compared with the model group.

We explored the influence of HJ on PEDV replication in Vero and IPI-FX cells at non-cytotoxic concentrations by qRT-PCR assays. As shown in Figure 2, compared with the model group the relative expression of PEDV N gene in the infected Vero or IPI-FX cells gradually decreased by increasing the concentration of HJ.

**FIGURE 3:**
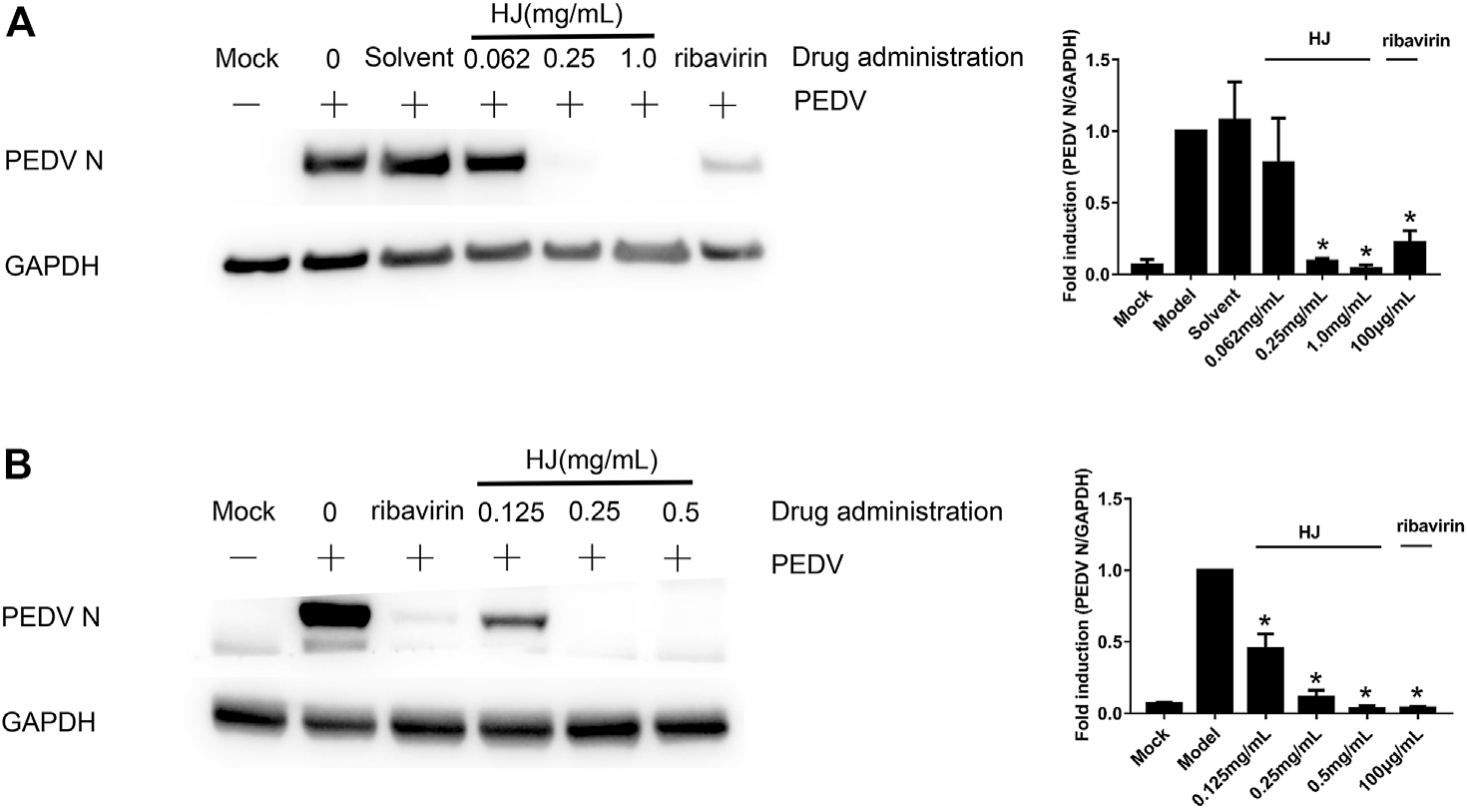
The expression level of PEDV N protein in Vero **(A)** or IPI-FX **(B)** cells were examined by western blot assays. Cells were treated with HJ throughout the experiment. The expression level of PEDV N protein was calculated in relation to the expression level of GAPDH at 24 hpi. Results are representative of three independent experiments. Data are represented as mean ± SD, *n* = 3. *, and *p* < 0.05 was considered statistically significant compared with the model group.

In addition, in order to further demonstrate the inhibitory effect of HJ on PEDV, the level of PEDV N protein in infected cells was detected at 24 h by western blot assays. As shown in Figure 3, the expression level of PEDV N protein both in infected Vero and IPI-FX cells was reduced by treatment with HJ. It is to be noted that no specific band was observed in infected cells when HJ reached the concentration of 0.5 mg/mL, indicating that PEDV may be completely removed.

**FIGURE 4:**
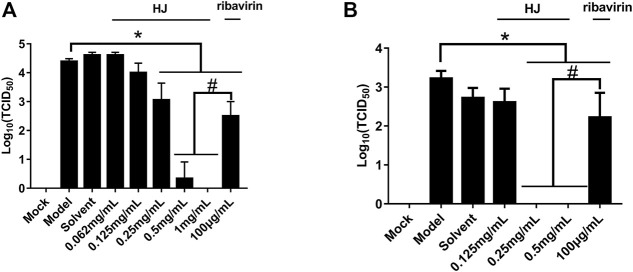
The viral titers of PEDV-CV777 **(A)** and PEDV-G2 **(B)** were determined by measuring TCID_50_ with the end-point dilution methods. Cells were treated with HJ throughout the experiment, and samples were harvested for 24 h. Data are represented as mean ± SD, n = 3. *, and *p* < 0.05 was considered statistically significant compared with the model group, while ^#^, and *p* < 0.05 was considered statistically significant compared with ribavirin group.

Furthermore, the virus titers of PEDV incubated with HJ were also detected in Vero and IPI-FX cells. As shown in Figure 4, when the concentration of HJ reached 0.5 mg/mL, the virus was completely inhibited, and these results were consistent with those of western blot. Taken together, HJ could inhibit PEDV replication *in vitro*.

**FIGURE 5:**
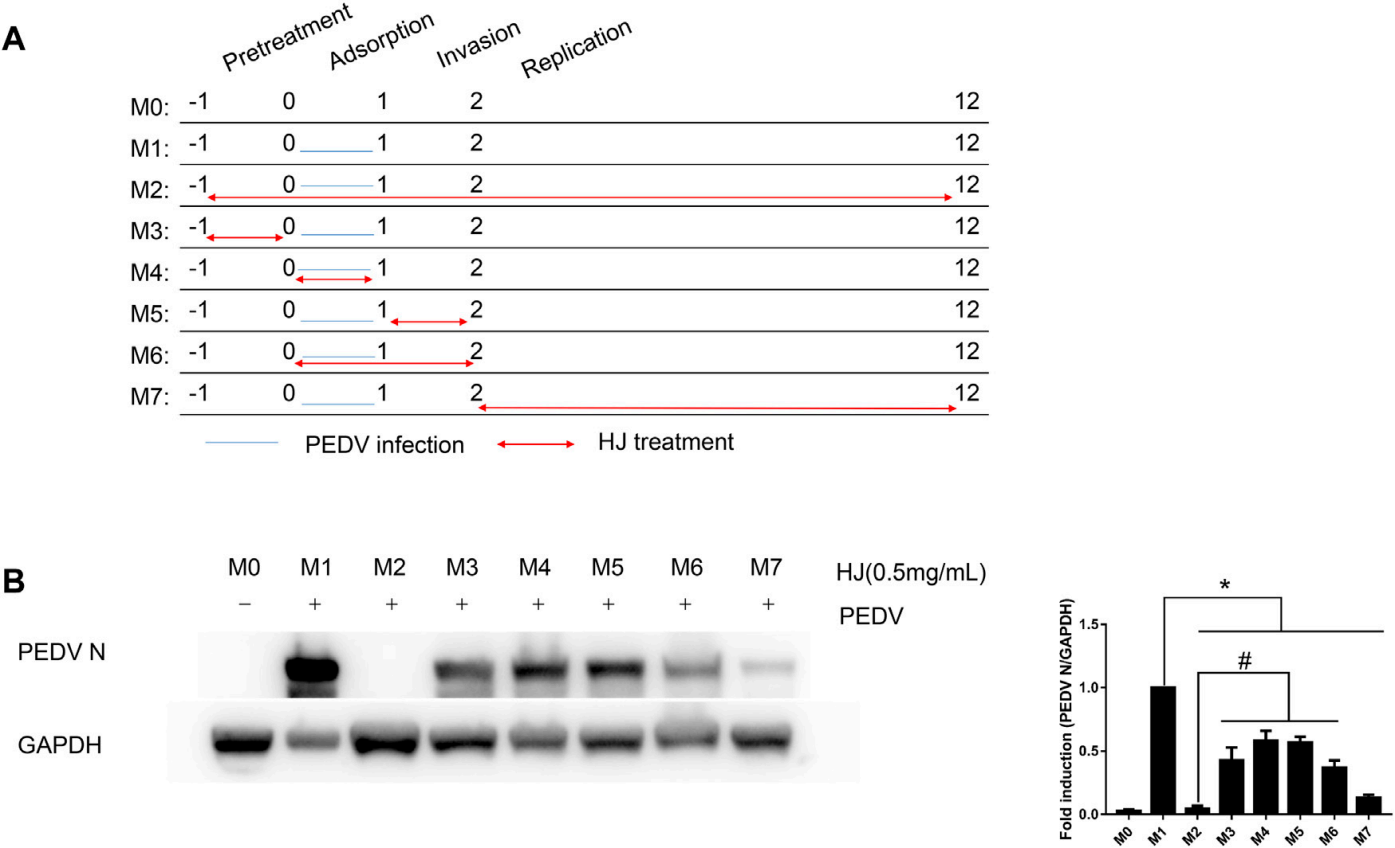
Inhibitory effects of HJ on IPI-FX cells infected with PEDV virus at different stages. **(A)** IPI-FX cells were treated with HJ at different times before or after infection. Double-headed red arrows indicate the presence of HJ, while the orange horizontal lines indicate the time of PEDV infection. The experiments are identified in the text by the numbers on the left (M0–M7). **(B)** The expression level of PEDV N protein in IPI-FX cells was examined by western blot assays. Cells were harvested for 12 h. The expression level of PEDV N protein was calculated in relation to the expression level of GAPDH. Results are representative of three independent experiments. Data are represented as mean ± SD, *n* = 3. *, and *p* < 0.05 was considered statistically significant compared with M1, while ^#^, and *p* < 0.05 was considered statistically significant compared with M2.

In order to determine the stage of the viral cycle where HJ exhibits its activity against PEDV, time of addition assays were carried out. As shown in Figure 5, compared with the M1 group,the expression level of PEDV N protein in M3 to M7 were all decreased indicating that HJ was effective in all time periods. Meanwhile, compared to the M2 group, only the M7 had no difference in the expression level of PEDV N protein, indicating that the inhibitory effect of HJ on PEDV was mainly in the replication stage. In addition, HJ also showed weak inhibitory effect during the prevention, viral adsorption, and viral invasion stages.

### 3.3 HJ protected piglets against PEDV infection *in vivo*

**FIGURE 6:**
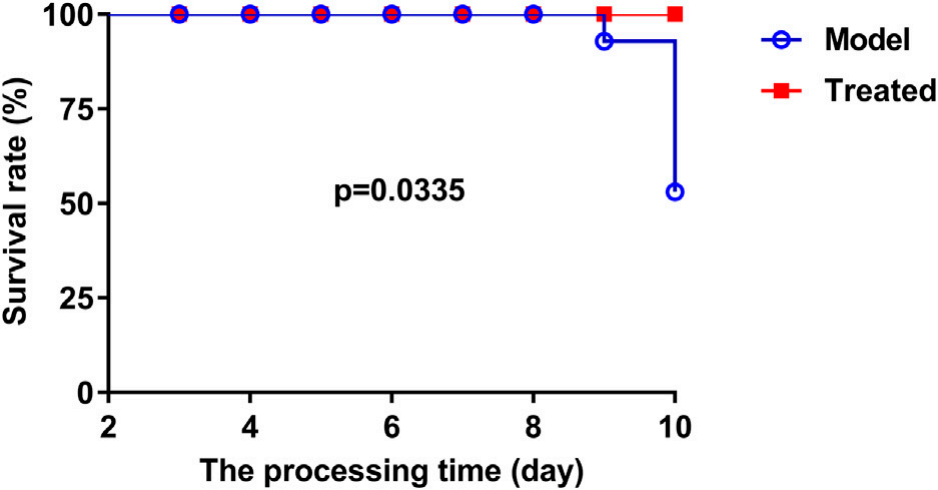
The effect of HJ on the survival rate of PEDV-challenged piglets was recorded daily. At 3 days of age, piglets were orally administered HJ or water twice a day. All piglets were orally challenged with 5 mL DMEM containing a total of l0^5^ PFU of PEDV-G2 solution on day 8.

**FIGURE 7:**
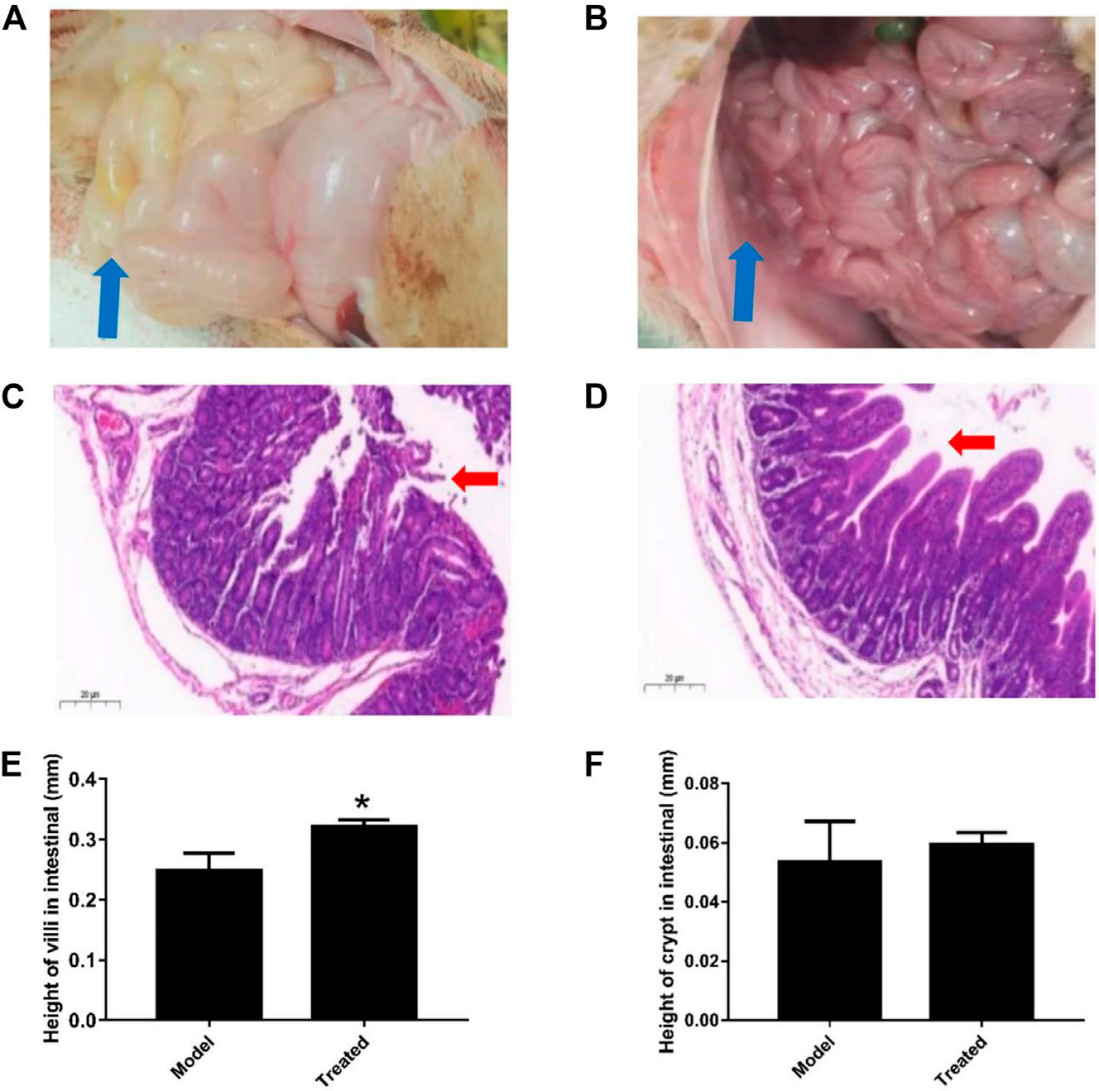
Effect of HJ on the macroscopic and histological changes of piglets infected with PEDV. Piglets received HJ (1.28 g/kg) by oral administration for 6 days before PEDV-G2 (5 mL DMEM containing l0^5^ PFU) infection. The intestinal microscopic lesions of piglets in the model group **(A)** and the treated group **(B)** were recorded before sampled. The thin-wall intestinal tracts containing undigested food were indicated by blue arrows. The histological changes of intestine of piglets in the model group **(C)** and the treated group **(D)** were analyzed by hematoxylin-eosin (HE) staining. The damage and shedding of intestinal villi were indicated by red arrows. The height of villi **(E)** and crypt **(F)** in HE staining were measured to evaluate the growth and development of intestinal. Data are represented as mean ± SD, *n* = 3. *, and *p* < 0.05 was considered statistically significant compared with the model group.

**FIGURE 8:**
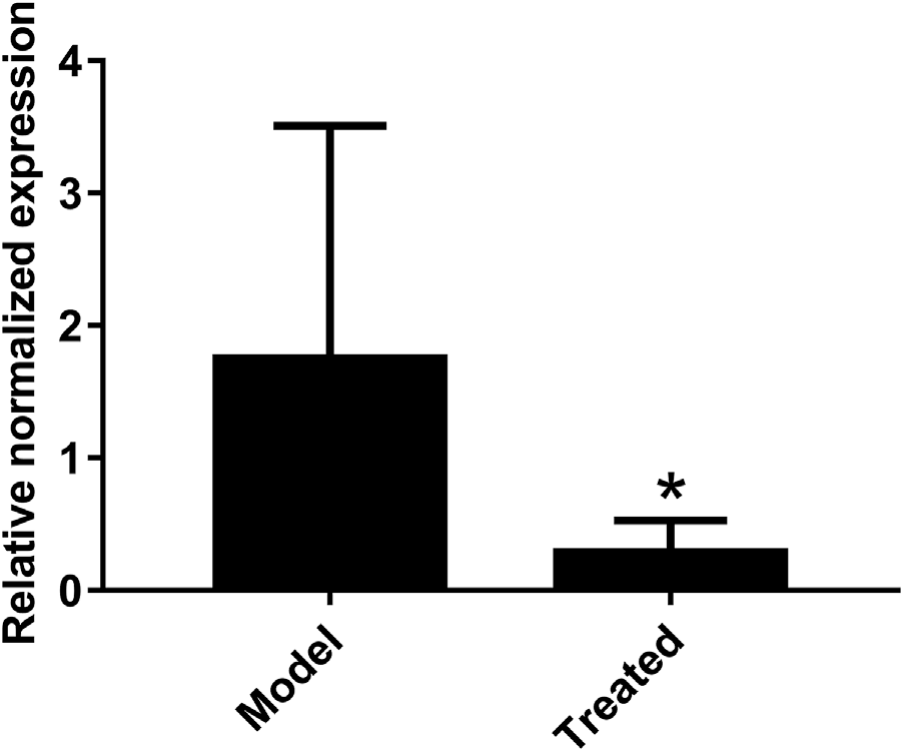
Effect of HJ on the viral load of PEDV in the ileum of piglets. The presence of PEDV in ileum were examined with real-time PCR using specific primers. The expression levels of mRNA were calculated in relation to the expression level of GAPDH. Data are represented as mean ± SD, *n* = 7. *, and *p* < 0.05 was considered statistically significant compared with the model group.

The dosage of *Hypericum japonicum* was determined based on the dosage in Chinese Veterinary Pharmacopoeia, and then whether HJ could protect piglets from PEDV attack was studied *in vivo*. All piglets were fed with milk powder manually to avoid the influence of diet on experimental results. The results showed that the model group (PEDV challenge group without HJ treatment) had severe symptoms characterized by acute vomiting and watery diarrhea, and three piglets died, while there was no death and less incidence of vomiting and diarrhea in the treated group. At 6 days before PEDV challenge, HJ was administered daily until the end of the experiment (Figure 6). To further study the gross pathological and histological changes of piglets infected with PEDV, the piglets in each group were dissected 48 h post infection. In the model group, a large amount of undigested milk and water was observed in the small intestine of infected piglets, and the intestinal wall of infected piglets was inflated with transparent thin wall, while no or slight lesions were observed in the treated group (Figure 7). Moreover, the results of microscopic lesions showed that the intestinal villi in the model group were severely atrophied, fractured, and shed, while the villi in treated group had a complete shape, clear contour, and regular arrangement. Specifically, the height of intestinal villi in the treated group was also significantly higher than that in the model group. In addition, the viral load of PEDV in the ileum was analyzed by qRT-PCR, and the results showed that the viral load of the HJ-treated group was lower than the model group (Figure 8). In conclusion, HJ had a certain protective effect against PEDV infection in piglets.

### 3.4 HJ treatment altered the intestinal microbiota of piglets

**FIGURE 9:**
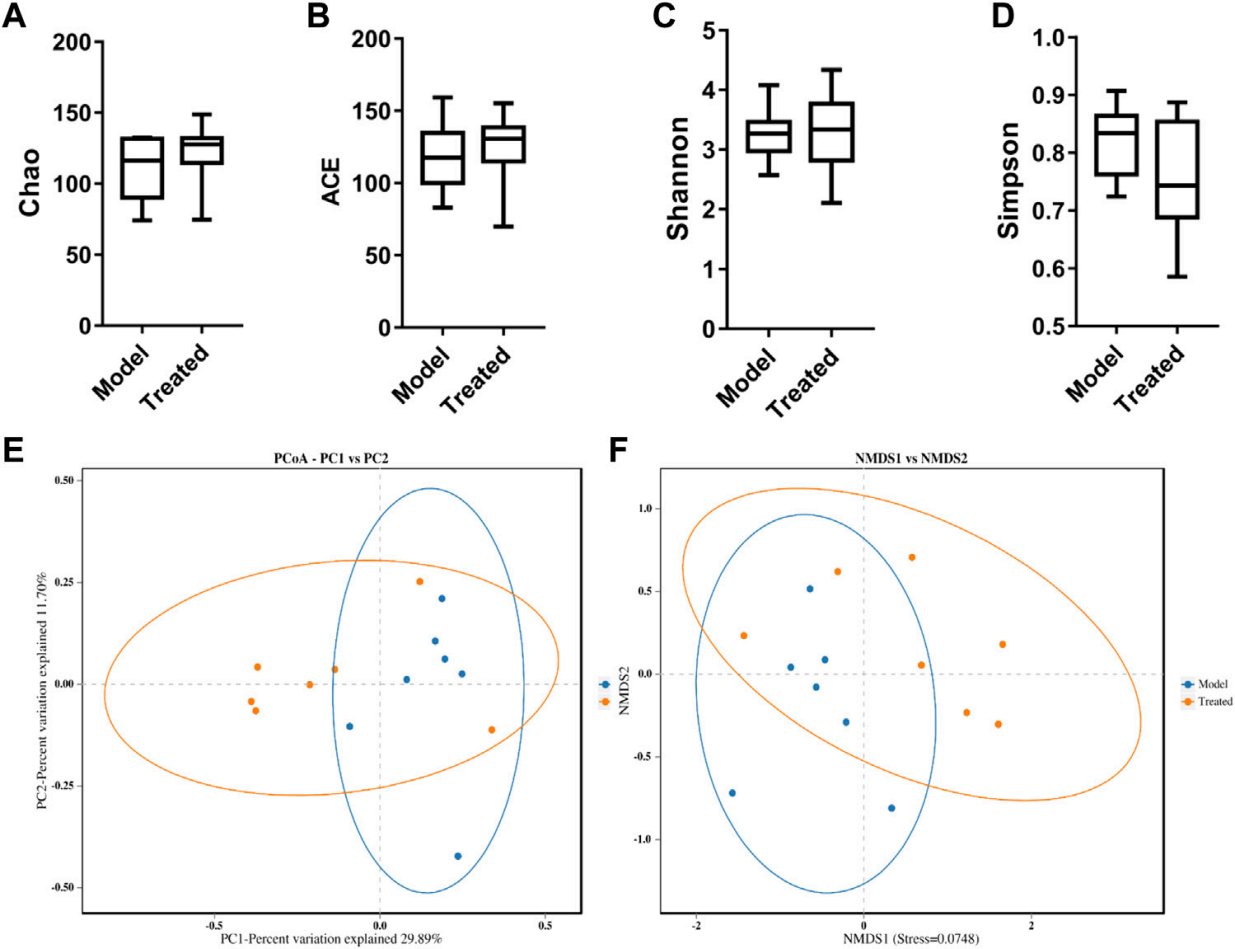
Alpha-diversity and beta-diversity analysis on the intestinal microbiota of piglets. **(A)** Chao, **(B)** ACE, **(C)** Shannon, and **(D)** Simpson indexes calculated after rarefying to an equal number of sequence reads for all samples. Data are represented as box plots. **(E)** PCoA of the intestinal microbiota of 10-day-old piglets in different groups. The blue dots represented the model group, and the orange dots represented the treated group. **(F)** NMSD of the intestinal microbiota of 10-day-old piglets in different groups. Model represents piglets challenged with PEDV, and treated represents piglets received an oral administration of HJ before PEDV challenged. Dots of the same color indicate that they belong to the same group. The closer the dots were, the more similar the composition of the samples was, *n* = 7.

**FIGURE 10:**
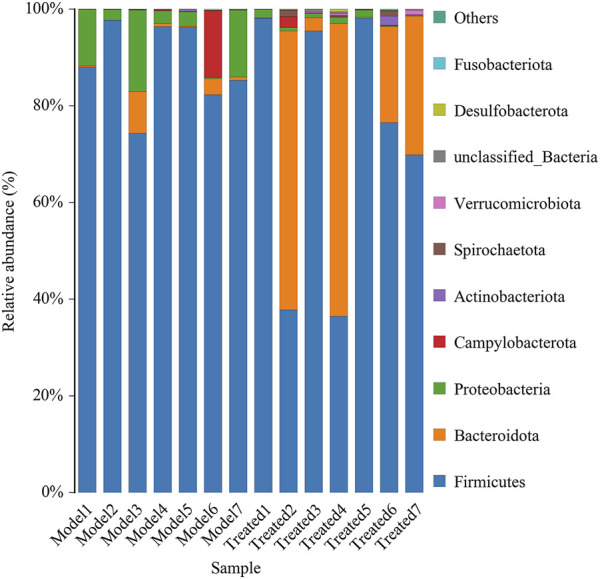
Average relative abundance of small intestinal microbial species at the phylum level. Model represents piglets challenged with PEDV, and treated represents piglets received an oral administration of HJ before PEDV challenged, *n* = 7.

**FIGURE 11:**
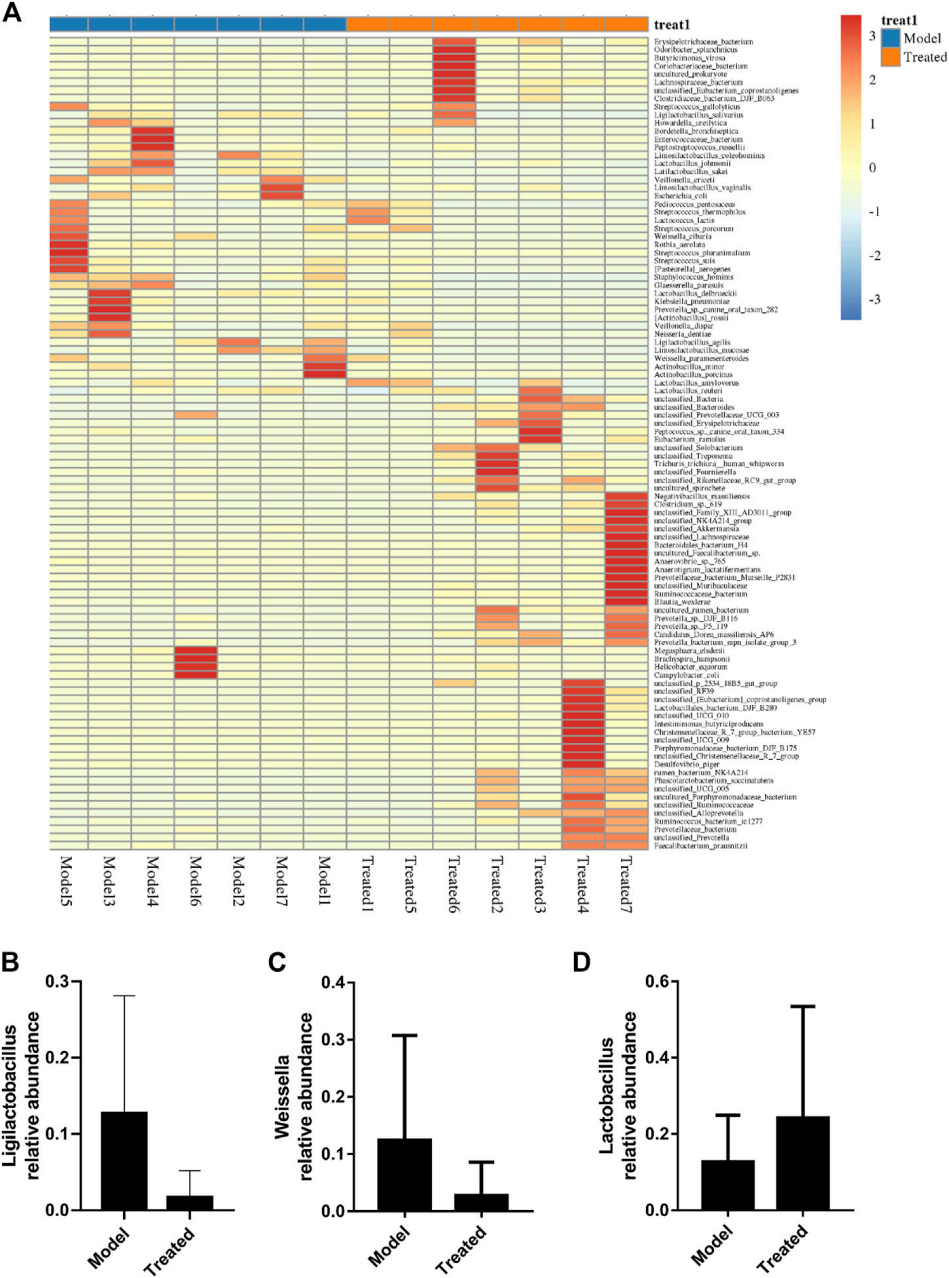
Effects of HJ on intestinal microbiota and key microbiota at the species level. **(A)** Species composition heat map at the genus level for species clustering. The higher the yellow degree was, the lower the relative abundance of the species was; and the higher the red degree was, the higher the relative abundance of the species was. The contents of **(B)** Ligilactobacillus_agilis, **(C)** Weissella_cibaria, and **(D)** *Lactobacillus*_amylovorus were measured to evaluate the changes of intestinal microbiota. Data are presented as mean ± SD, *n* = 7.

Intestinal microbiota is a stable microenvironment in the whole intestine, which plays a vital role in the suppression of pathogenic bacteria and the development of intestinal mucosal immune system. In the present study, a total of 14 intestinal content samples was collected from piglets and subjected to 16S rRNA sequencing. After screening of the original sequence reads, 173,529 valid reads in the V3–V4 region of 16S rRNA were obtained. The average length of the amplicon was 462 bp. The lengths of the effective sequences ranged from 1,470 bp to 1,494 bp, with an average length of 1,485 bp. A total of 252 OTUs was classified into different taxonomies. Specifically, 12 phyla, 19 classes, 35 orders, 63 families, 126 genera, and 180 species were identified from these samples based on sequence clustering. To investigate how HJ affected the richness and diversity of intestinal microbiota, the index of Chao, Ace, Shannon, and Simpson were calculated to estimate the alpha diversity, while the PCoA analysis and NMDS analysis were performed to estimate the beta diversity. Shannon and Simpson indices were quantitative measures of bacterial diversity, reflecting both species richness and evenness. The larger the Chao, ACE, and Shannon index were; and the smaller the Simpson index was; indicating a richer sample species was. As shown in Figure 9, the aforementioned indexes of each group showed that the abundance and diversity of intestinal microbiota of piglets in HJ administration group were not significantly different from those in the model group, but the composition of intestinal microbiota was different in the two groups, suggesting that HJ changed intestinal microbiota of piglets by regulating the structure of microbiota rather than species diversity. Furthermore, the HJ administration group increased the abundance of Bacteroidetes and decreased the abundance of Proteobacteria in PEDV-infected piglets at the phylum level (Figure 10). At the species level, HJ have a tendency to decrease the abundance of Ligilactobacillus_agilis and Weissella_cibaria, and increase the abundance of Lactobacillus_amylovorus, but no significant differences were found (Figure 11).

## 4 Discussion

PEDV variants have spread rapidly in China and throughout the world in recent years, causing significant problems to the swine industry as its infections become more widespread (Xie et al., 2021; He et al., 2022). The invasion of pig intestinal epithelial cells by PEDV can cause enteritis in pigs of all ages and often lead to death of newborn piglets (Wen et al., 2018). Although vaccines against PEDV exist, they are not optimal treatments in terms of safety and efficacy, resulting in large-scale infections still occurring (Shi et al., 2019). Therefore, it is imperative to find new drugs against PEDV. Herbal medicines have been popular for screening new antiviral agents due to their low costs, low side effects, and high availabilities (Kim et al., 2015; Ali et al., 2021; Huan et al., 2021). In the present study, we reported that *Hypericum japonicum*, an inexpensive and widely distributed traditional Chinese herbal medicine, had an inhibitory effect on PEDV infection both *in vitro* and *in vivo*, which may be helpful for the control of PED in pig farms.

Since the cytotoxicity of HJ may affect the judgment of the antiviral effect *in vitro*, we conducted the cytotoxicity analysis of the drug before the antiviral test. In this study, the CCK-8 assay was chosen to determine the maximum non-cytotoxic concentration of HJ on Vero and IPI-FX cells because these cell types were commonly used in the study of PEDV (Huan et al., 2017; Wang X. et al., 2019). Ribavirin is a broad-spectrum antiviral drug that exhibits significant inhibitory activity on a variety of RNA and DNA viruses (Kali et al., 2021). In this study, ribavirin, which was used as a positive comparator, also showed a good inhibitory effect on PEDV. As is known to all, drug resistance is one of the key problems in antiviral drug research studies (Delang et al., 2014; Dagher et al., 2017). Interestingly, 0.5 mg/mL of HJ could completely inhibit PEDV both in viral titer and PEDV N protein, which indicated that HJ had the potential to reduce the development of drug resistance in PEDV.

In order to investigate the stage at which HJ exhibted its antiviral effect in the PEDV infection process, we first explored the effect of time of addition assay, and the result suggested that HJ exhibited its inhibitory effect in the entire stage of viral replication with a focus on the replication stage. It has been reported that quercetin 7-rhamnoside, a flavonoid abundant in *Hypericum japonicum*, exhibited its inhibitory effect at the early stages of viral replication (Song et al., 2011). Quercetin, another flavonoid in *Hypericum japonicum*, could inhibit the activity of PEDV 3CLpro by binding to the active sites and S1 specificity pocket of PEDV 3CLpro (Li et al., 2020). Therefore, the anti-PEDV effect of *Hypericum japonicum* might be related to the flavonoids it contains, thus inhibiting the active protease 3CLpro of the virus (Peron et al., 2019).

PEDV has been reported to be most harmful to suckling piglets between 7 and 10 days of age, with high mortality reaching 90% (Li et al., 2018). In this study, newborn piglets were fed with a safe dose of HJ and then infused with PEDV. Compared with the model group, the mortality of piglets treated with HJ was decreased; the diarrhea symptoms were alleviated; the intestinal villus damage was reduced; and the content of virus in the ileum was lower, which confirmed that HJ could significantly protect piglets from PEDV infection *in vivo*.

With the development of sequencing technology, the increasing number of studies has revealed a close relationship between gut microbiota and intestinal health (Liu et al., 2017). More and more probiotics, such as *Lactobacillus*, have been directly or indirectly used in the treatment of piglet diarrhea (Huang Z. et al., 2018; Dong et al., 2021). In this study, we used high-throughput sequencing of the V3 and V4 region of 16S rRNA gene, alpha-diversity analysis and beta-diversity analysis to investigate the effect of *Hypericum japonicum* on intestinal microbiota in piglets after PEDV challenge. Studies have shown that PEDV can cause the promotion of Firmicutes (Song et al., 2017; Yang et al., 2020; Wu et al., 2021). Meanwhile, the intestinal microbiota after HJ treatment increased the abundance of Bacteroidetes and decreased the abundance of Firmicutes and Proteobacteria suggesting that HJ might have the effect of restoring the intestinal microbiota composition of piglets after PEDV challenge. Furthermore, HJ had a tendency to reduce the abundance of Ligilactobacillus_agilis and Weissella_cibaria, which might be related to intestinal inflammation, and increase the abundance of *Lactobacillus*_amylovorus, which was resistant to PEDV infection (Zhang et al., 2019; Huang et al., 2020; Chen et al., 2022). Thus, HJ might play a protective role in PEDV-induced injury by regulating intestinal microbiota.

In conclusion, this research reported, for the first time, the characteristics of the HJ against PEDV. Our research demonstrated that HJ had antiviral activity against PEDV infection both *in vivo* and *in vitro* and could also inhibit piglet diarrhea by regulating the intestinal microbiota of piglets, which provided a possibility for the development of a new antiviral drug for the prevention and treatment of PEDV in pig farms. However, there are still several important problems to be solved. For example, what key compounds in *Hypericum japonicum* are responsible for inhibiting PEDV replication? What is the exact underlying mechanism? These questions will be explored in the near future, and elucidation of these will contribute to the development of safe and effective antiviral drugs to control porcine coronavirus.

## Data Availability

The datasets presented in this study can be found in online repositories. The names of the repository/repositories and accession number(s) can be found in: Sequence Read Archive (SRA), with the accession number PRJNA917445.
