# Supplementary material for: Hypericum japonicum extract inhibited porcine epidemic diarrhea virus in vitro and in vivo
Source: Front Pharmacol. 2023 Apr 17;14:1112610. doi: 10.3389/fphar.2023.1112610 (PMC10149974; doi:10.3389/fphar.2023.1112610)
Supplement: Supplementary file 1 [file DataSheet1.docx]

Supplementary Material

**Supplementary Figure 1.** The figure legends are required to have the same font as the main text, 12 point normal Times New Roman, single spaced. Please use a single paragraph for each legend and prepare the figures keeping in mind the PDF layout.


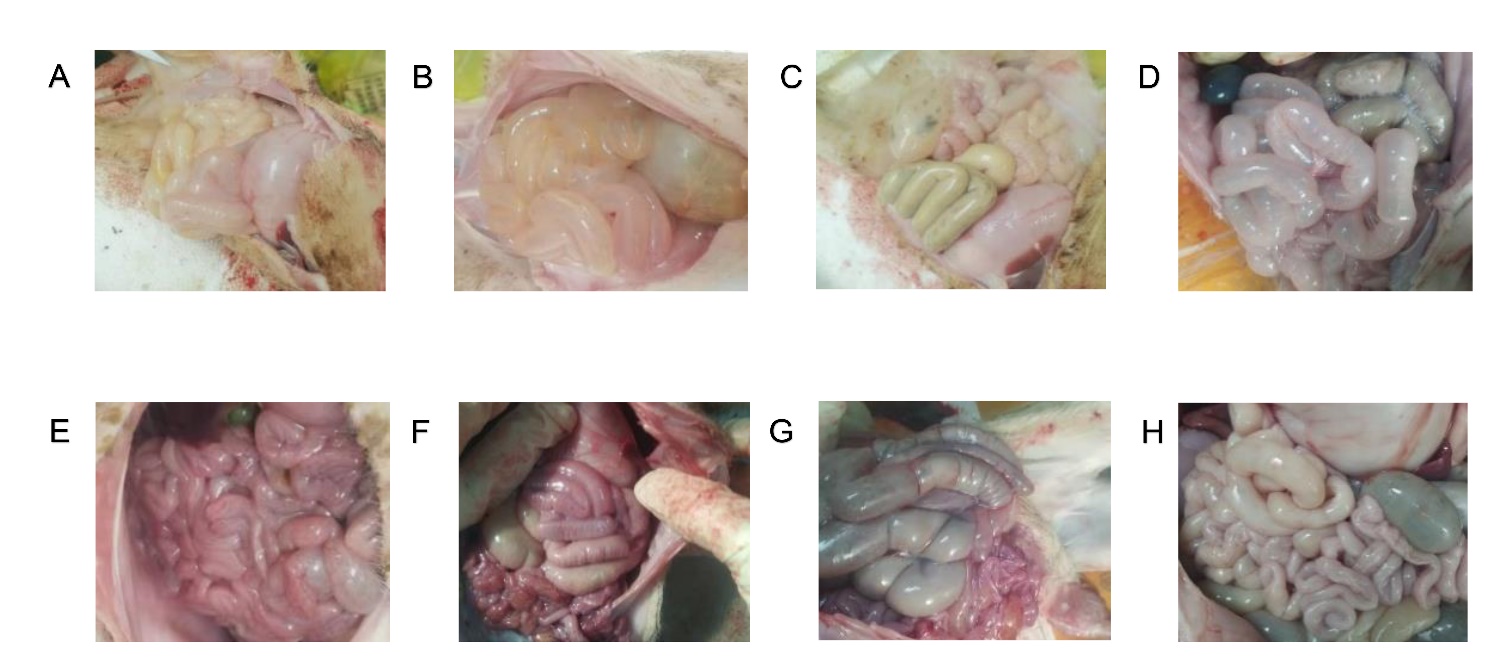


Fig.S1 Effect of HJ on the macroscopic changes of piglets infected with PEDV. Piglets were received HJ (1.28g/kg) by oral administration for 6 days before PEDV-G2 (5mL DMEM containing l0^5^ PFU.) infection. The intestinal microscopic lesions of piglets: (A-D) the model group, (E-F) the treated group. All pictures are from different piglets.


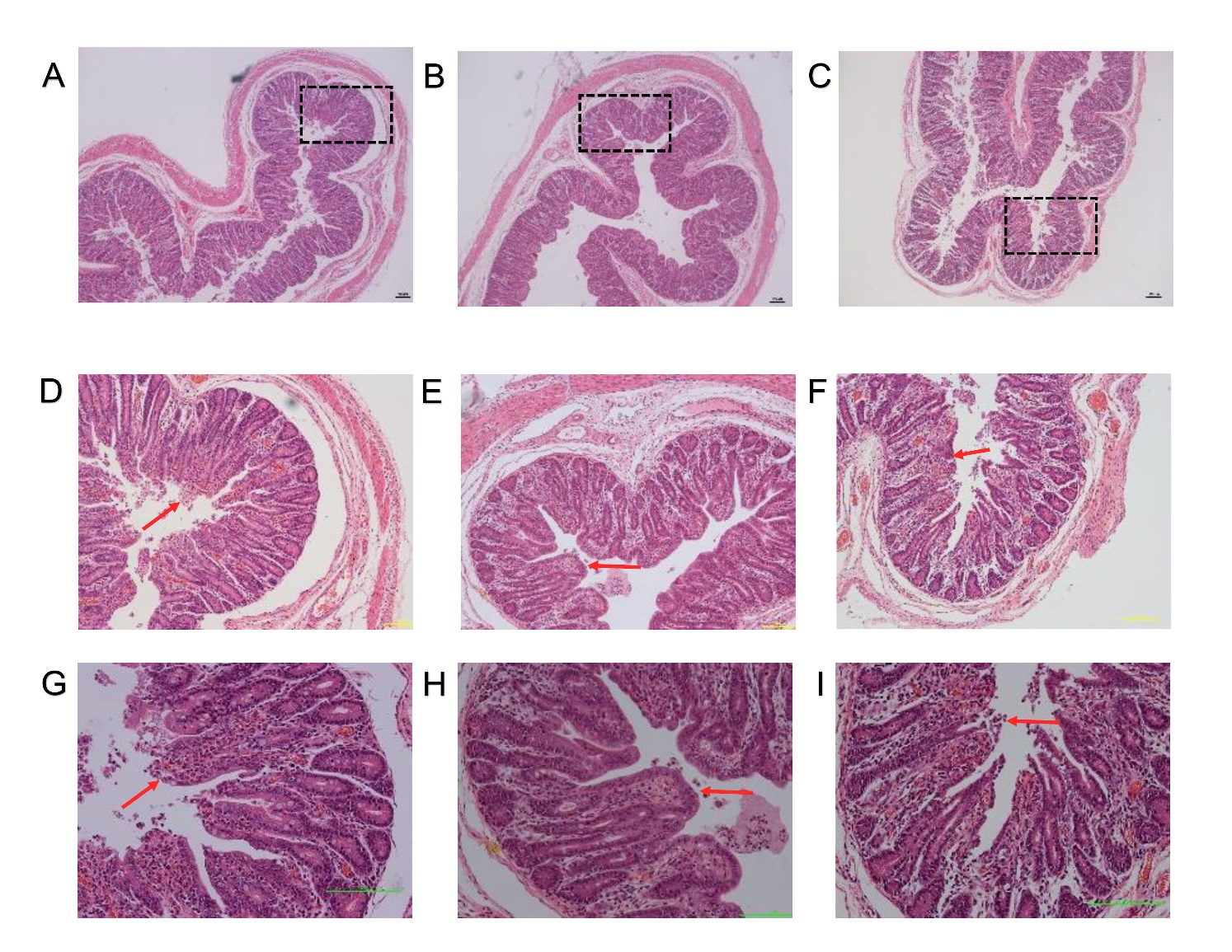


Fig.S2 Histological changes of piglets infected with PEDV. Piglets were infected with PEDV-G2 (5mL DMEM containing l0^5^ PFU.). The damage and shedding of intestinal villi were indicated by black box and red arrows. Bars=100 μm for all samples.


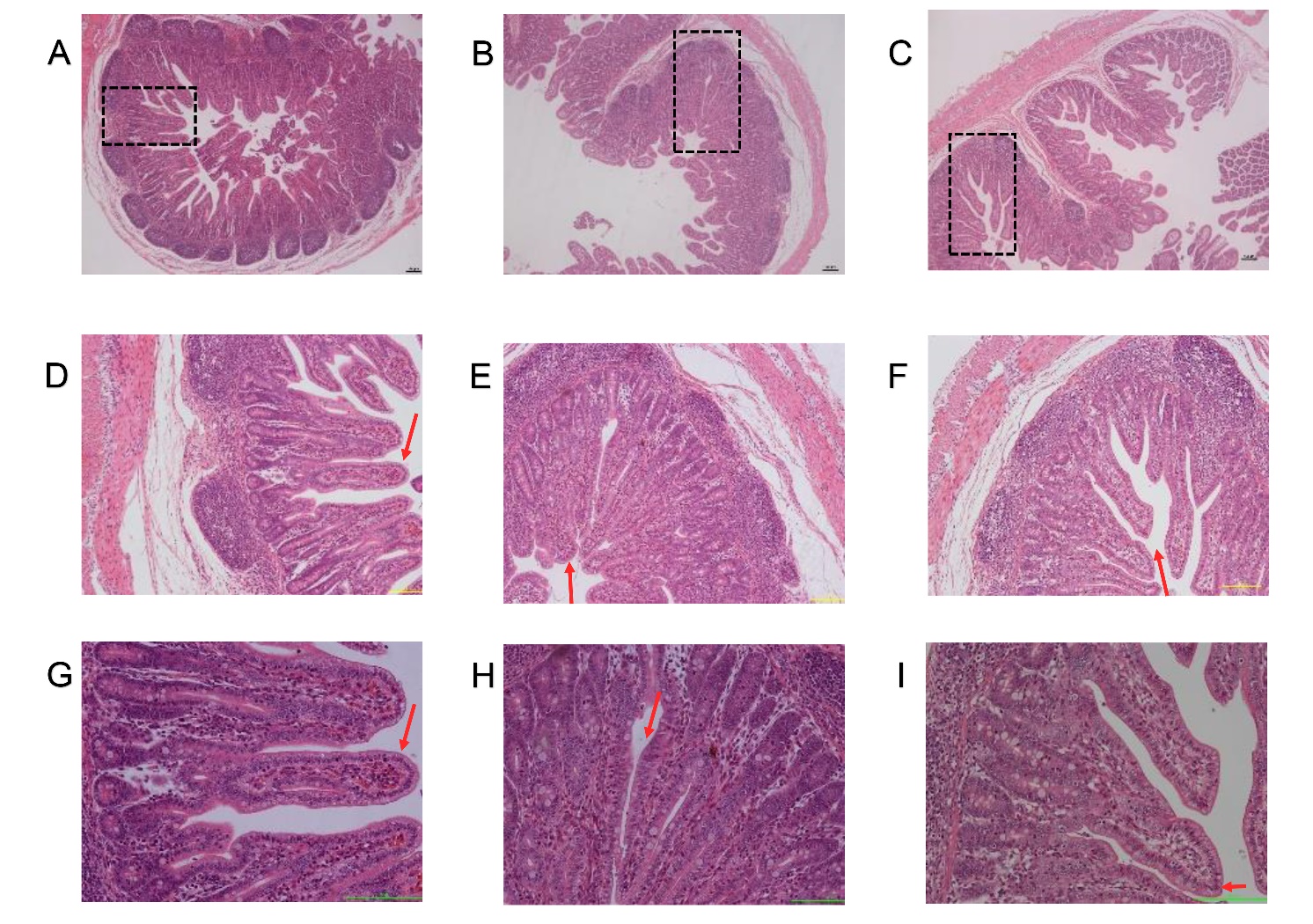


Fig.S3 Effect of HJ on the histological changes of piglets infected with PEDV. Piglets were received HJ (1.28g/kg) by oral administration for 6 days before PEDV-G2 (5mL DMEM containing l0^5^ PFU.) infection. The structurally intact of intestinal villi were indicated by black box and red arrows. Bars=100 μm for all samples.
